# Natural Teeth for Prosthetic Solutions in Pediatric Patients: Two Case Reports of Biologically Based Removable Functional Partial Dentures

**DOI:** 10.7759/cureus.69921

**Published:** 2024-09-22

**Authors:** Debapriya Pradhan, Saurabh Tiwari, Nikita Saini, Aishwarya Jethi, Khushboo Singh

**Affiliations:** 1 Department of Pedodontics and Preventive Dentistry, Hitkarini Dental College and Hospital, Jabalpur, IND

**Keywords:** clinical dentistry, clinical pediatric dentistry, dentistry, denture aesthetics, immediate denture, natural teeth, pediatric, pediatric preventive dentistry, removable partial denture, space maintainer

## Abstract

Prosthetic appliances are essential for promoting the proper development of teeth, dental arches, and facial bones in children. Due to the constantly changing nature of a child’s oral environment, these appliances must be temporary and require regular maintenance and adjustment to avoid impeding orofacial growth. A fundamental aspect of contemporary dentistry is re-establishing aesthetics. The absence of front teeth can cause appearance-related concerns in children. Personal appearance holds significant importance for children, particularly during adolescence. Even preschoolers, aged three to five, begin to cultivate a keen sense of body image and are increasingly aware of how they are perceived by both their peers and adults. We executed two cases in which natural teeth were used as replacements for artificial teeth in partial dentures. In the first case, we used the exfoliated natural teeth in a removable partial denture for aesthetic rehabilitation. This method leverages the natural tooth’s inherent aesthetic qualities, proving especially valuable in the immediate replacement of anterior teeth, where appearance is critical. In another case, we utilized the natural teeth for the posterior region not only to enhance the patient’s chewing efficiency but also to facilitate the eruption of unerupted succedaneous teeth. Additionally, using the patient's own teeth helps to achieve proper occlusion and comfort after the denture is placed, thus ensuring that patient expectations are met in both situations.

## Introduction

During childhood and adolescence, the orofacial system is rapidly developing, making the replacement of missing teeth for space maintenance crucial. Prosthetic solutions must support natural growth, ensuring proper chewing, aesthetics, and speech while preserving oral tissues. This approach sets the stage for successful restorations in adulthood [1]. Various prosthetic options exist, including removable dentures, overdentures, fixed prostheses, and implants. For children and adolescents, removable partial and complete dentures with acrylic bases are commonly chosen as they can be easily adjusted to accommodate growth [2]. Over the past 10 years, interim denture techniques have seen remarkable progress. They are designed to preserve both appearance and function following tooth extraction, enabling patients to maintain their social lives without experiencing edentulism [3]. In children, they can also serve as temporary removable space maintainers. While clinicians are skilled in replacing missing teeth with artificial options, the impact of natural teeth on a patient’s overall appearance and personality is significant. This article describes two case reports crafting a removable prosthesis that incorporates the patient’s own natural tooth to fill a gap. This approach not only enhances aesthetics and boosts the patient's confidence but also is effective in maintaining space for and supporting the eruption of succedaneous teeth.

## Case presentation

Case 1

**Figure 1:**
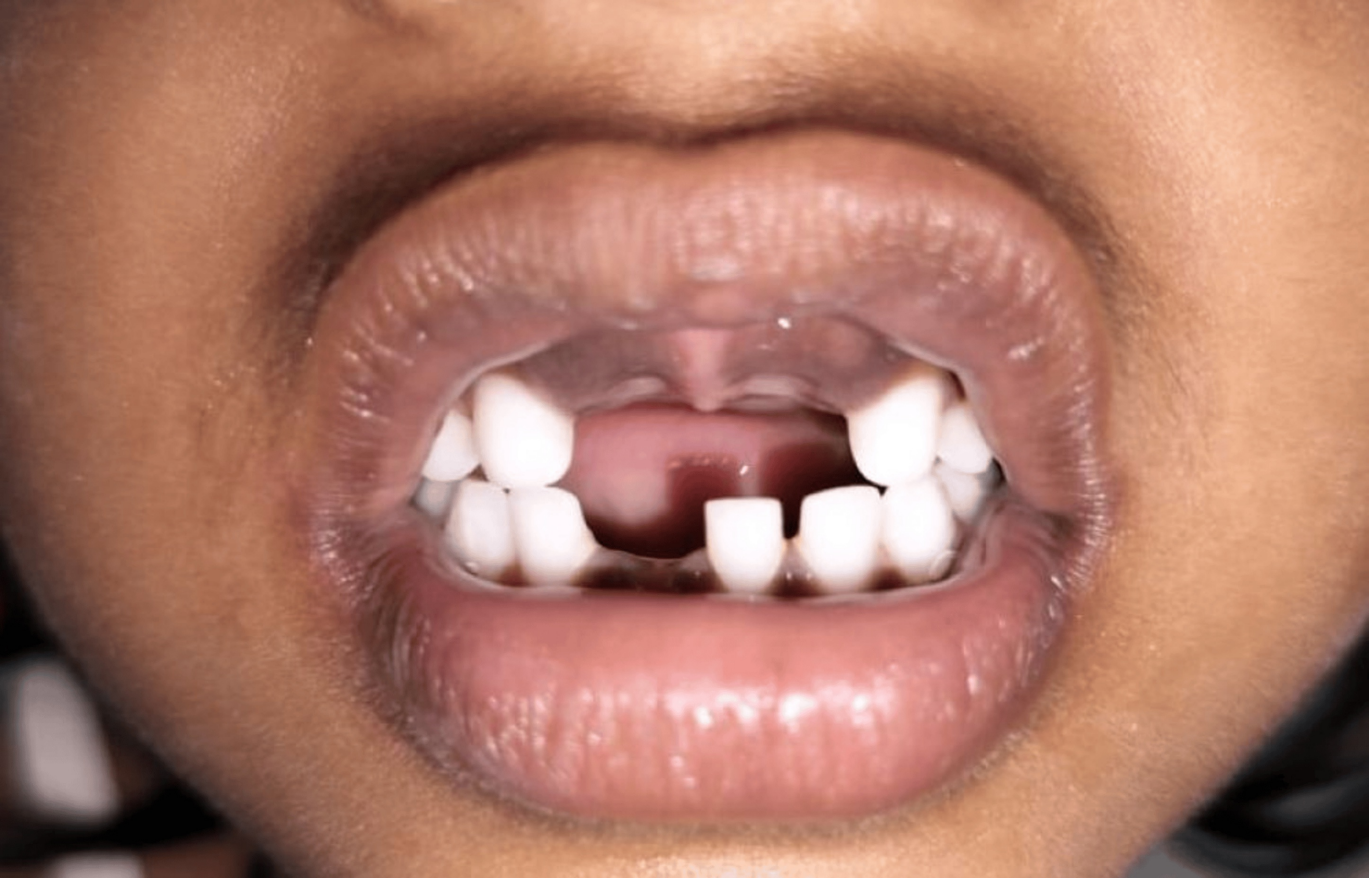
Pre-operative photograph illustrating the missing teeth in the anterior region.

A seven-year-old girl visited the Department of Pediatric and Preventive Dentistry at Hitkarini Dental College and Hospital with missing upper front teeth since one week ago (Figure 1).

**Figure 2:**
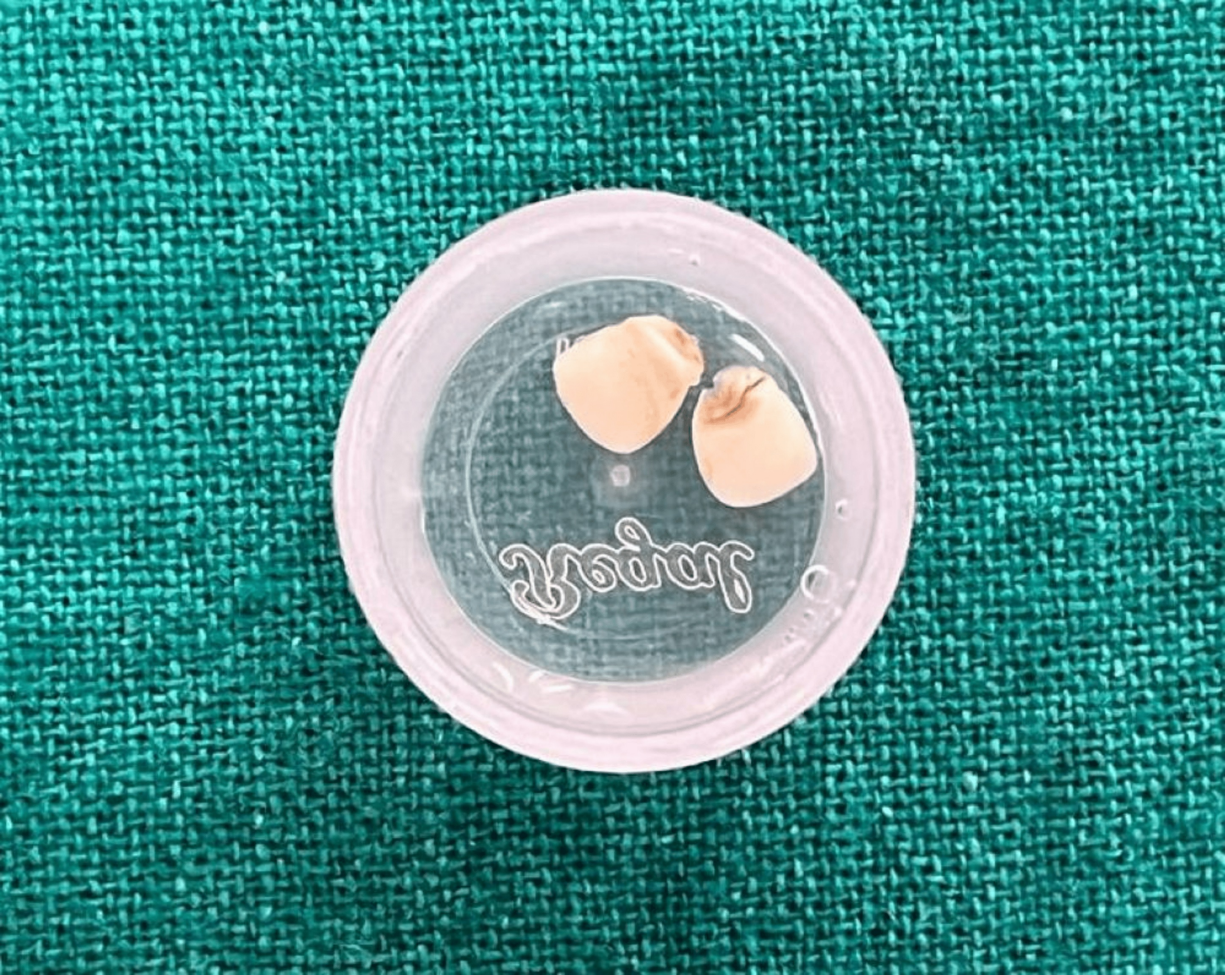
Patient's natural teeth (51 and 61) preserved in saline by the parents for one week.

Her parents reported that the teeth had fallen out after she repeatedly applied pressure to them with her fingers when they were already loose. The patient had no significant medical history. The child was anxious about her appearance due to an upcoming family event, prompting her parents to seek immediate temporary aesthetic restoration. Following advice from a local doctor, they stored the exfoliated teeth in saline (Figure 2).

Upon clinical examination, teeth 51, 61, and 81 were found to be missing, and the sockets had healed. Teeth 51 and 61 had been lost due to digit pressure, while tooth 81 had naturally exfoliated a few months earlier. Concerned about their daughter’s appearance, the parents requested an immediate temporary replacement using the stored teeth. A removable functional prosthesis for the maxillary arch, using the patient’s own teeth, was proposed.

**Figure 3:**
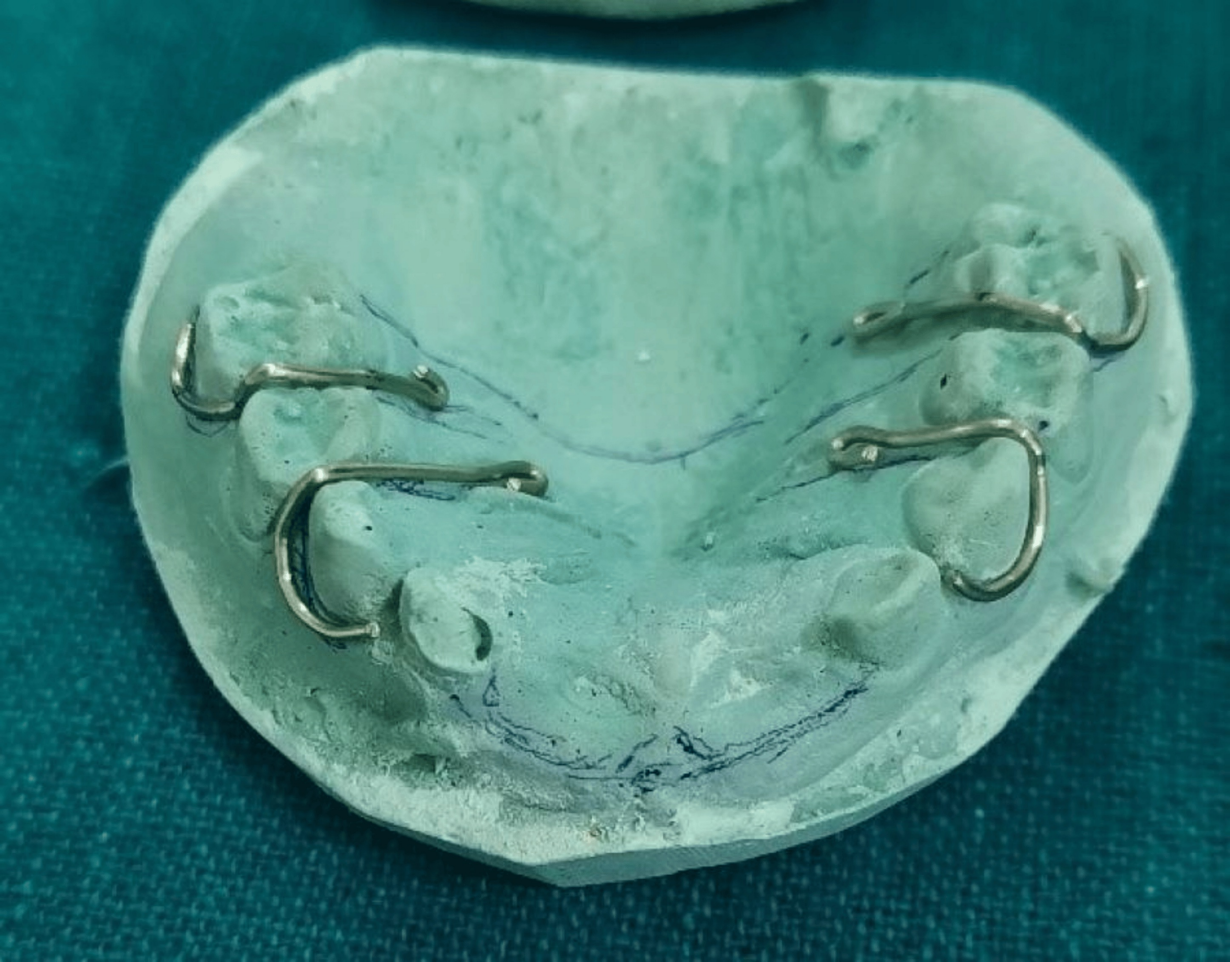
C-clasps fabricated on the cast for teeth 53, 55, 63, and 65.

After obtaining written consent from the parents, alginate impressions of both arches were taken, and diagnostic casts were created. The exfoliated teeth were cleaned using an ultrasonic scaler. With the roots mostly resorbed, the surfaces were smoothed down to the cemento-enamel junction. The coronal portion of each tooth was evened out at the cervical end, and a small cavity was created to access the pulp chamber. The pulp debris was removed, and the chambers were thoroughly irrigated with saline. The chambers were then etched with gel for one minute, followed by the application of a bonding agent. After light curing, the chambers were filled with flowable composite to completely seal the teeth. The natural teeth were then trimmed and adjusted to fit the alveolar bone flange. C-clasps were designed on the cast for teeth 53, 55, 63, and 65 to ensure stability (Figure 3).

**Figure 4:**
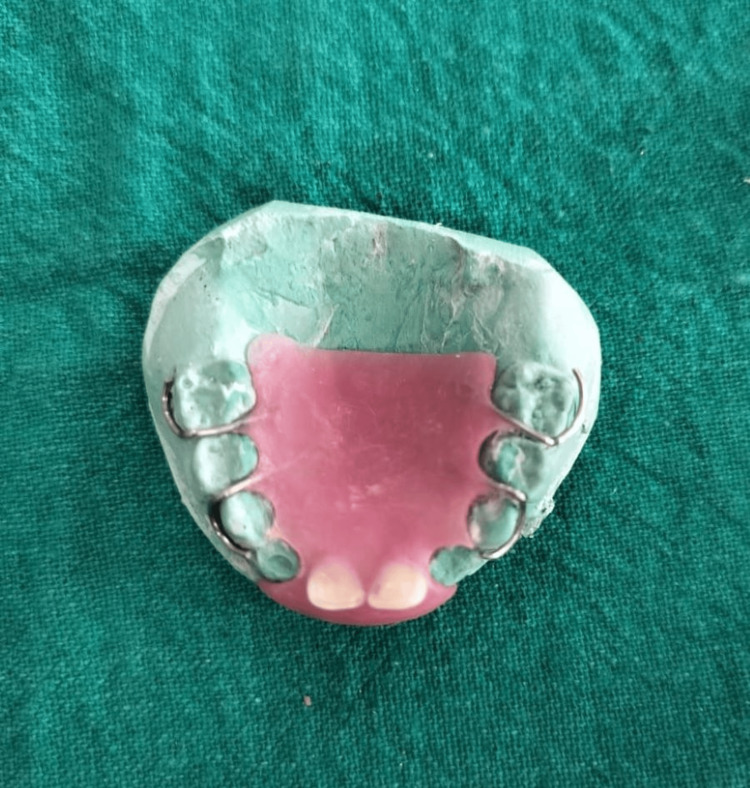
Removable partial denture fabricated with self-cure acrylic resin using natural teeth as pontics.

The palatal cervical regions of the teeth were contoured to ensure correct positioning on the cast. A biological removable partial denture was then fabricated using self-cure acrylic resin (Figure 4).

**Figure 5:**
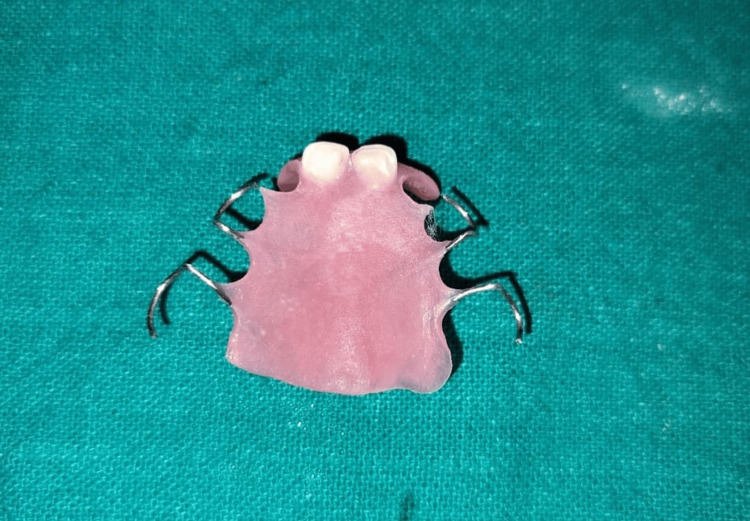
Trimmed, finished, and polished appliance.

Finishing and polishing of the appliance were done (Figure 5).

**Figure 6:**
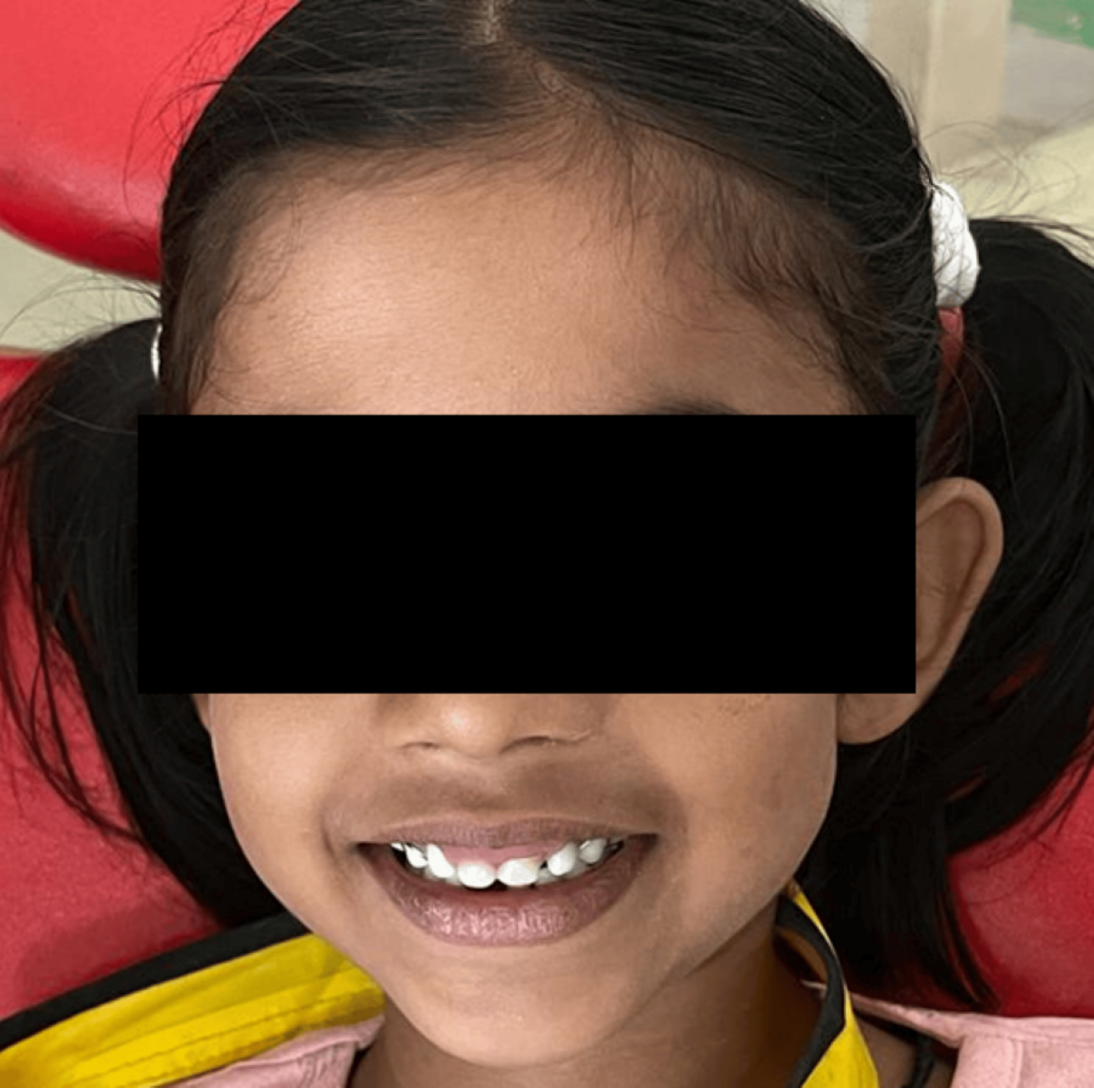
Photograph taken post-appliance delivery.

On the very same day, the appliance was delivered to the patient (Figure 6).

Instructions were provided on how to insert and remove the partial denture, and the patient and her parents were educated on proper oral hygiene. They were advised to brush the denture daily with a soft-bristled brush and mild soap to remove food deposits and plaque and to store the denture in water at night. Parents were informed that there might be some initial challenges with denture care. Natural teeth tend to keep their color and vibrant appearance when they remain moist. However, if they dry out, their appearance might temporarily change, though it generally returns to its original state once moisture is restored. After 24 hours, the denture was retaining well. Guardians were advised to bring the child in for regular dental check-ups to monitor the eruption of permanent teeth, as the denture could potentially interfere if not properly observed. The post-operative period was uneventful, and the child adapted quickly to the denture. The denture was removed when signs of the permanent incisors' eruption became apparent.

Case 2

**Figure 7:**
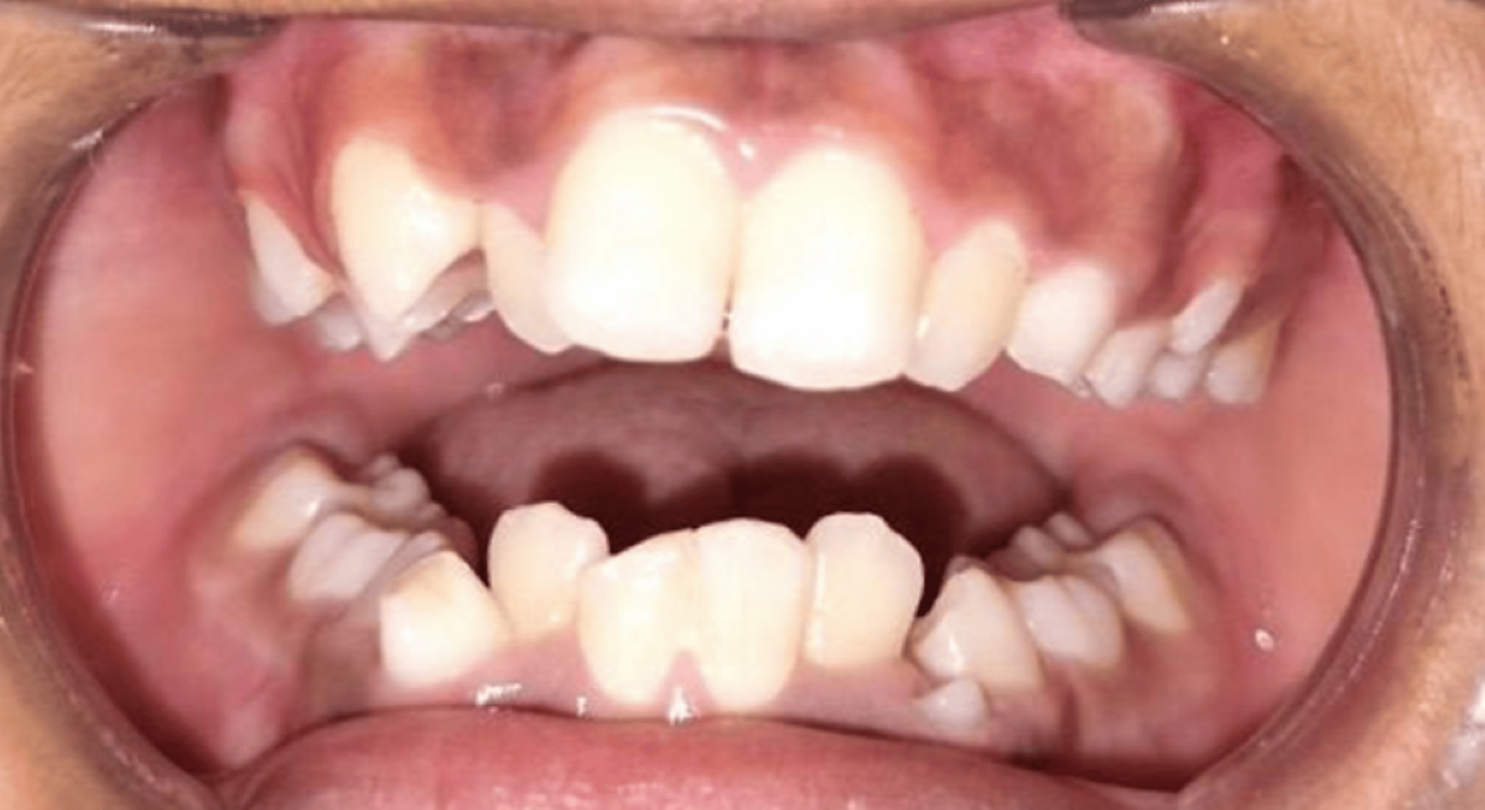
Pre-operative photograph demonstrating malaligned teeth.

A 13-year-old male patient presented to the Department of Pediatric and Preventive Dentistry at Hitkarini Dental College and Hospital with a primary concern of irregular teeth, seeking treatment for the condition (Figure 7).

**Figure 8:**
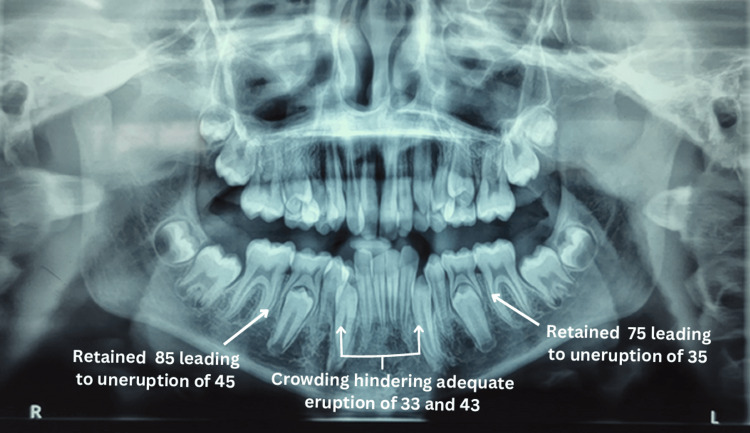
Photograph of orthopantomogram illustrating retained teeth 75 and 85 leading to the non-eruption of 35 and 45 as well as anterior crowding, thus leaving minimal space for the proper eruption of lower permanent canines.

The patient had no notable family or medical history. Clinical examination revealed anterior crowding and unerupted permanent teeth, primarily due to retained teeth 75 and 85. An orthopantomogram was recommended (Figure 8).

**Figure 9:**
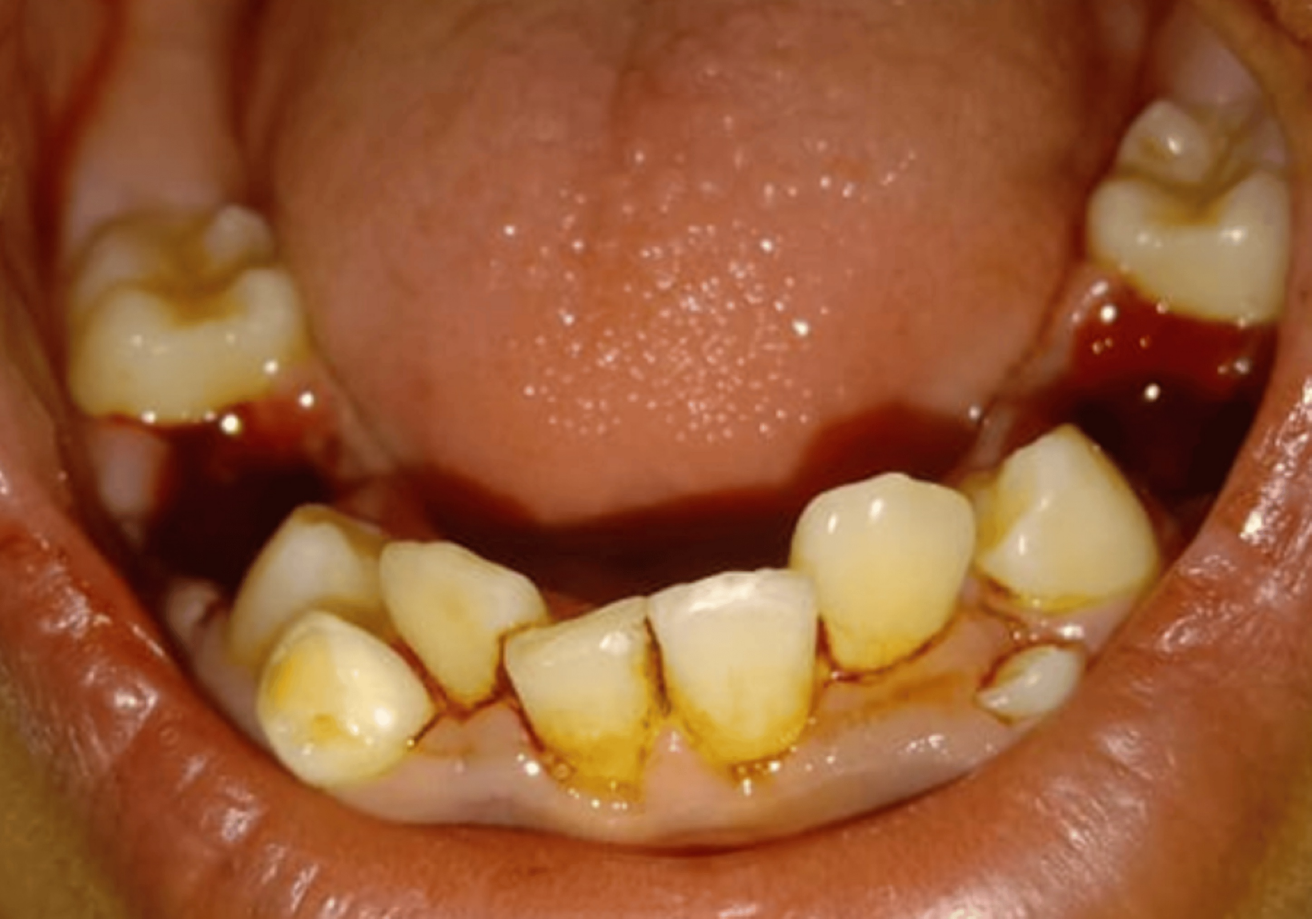
Photograph taken post-extraction in relation to teeth 75 and 85.

Radiographic examination revealed an absence in the resorption of the roots of both mandibular deciduous second molars, indicating that the eruption process of the succedaneous teeth had not yet begun. Therefore, prior to initiating fixed orthodontic treatment, it was decided to extract teeth 75 and 85 and place a biological removable functional space maintainer to encourage the eruption of teeth 35 and 45. The extracted molars were planned to be used as pontics. With written consent from the parents, alginate impressions of both arches were taken on the first day, and diagnostic casts were created. Teeth 75 and 85 were then extracted under local anesthesia (Figure 9).

**Figure 10:**
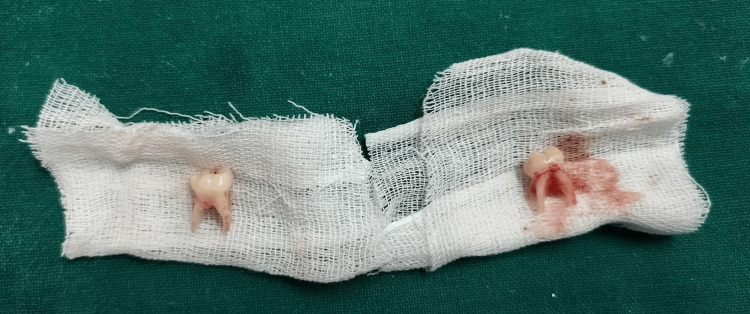
Extracted mandibular second deciduous molars.

The extracted teeth had well-preserved roots with no signs of resorption (Figure 10).

**Figure 11:**
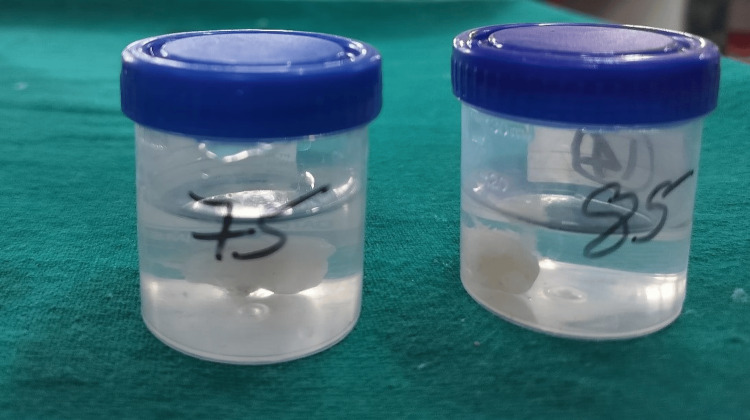
Extracted teeth 75 and 85 cleaned and preserved in saline.

The extracted second deciduous molars were thoroughly cleaned with water and a 3% sodium hypochlorite solution, then stored separately in saline (Figure 11).

**Figure 12:**
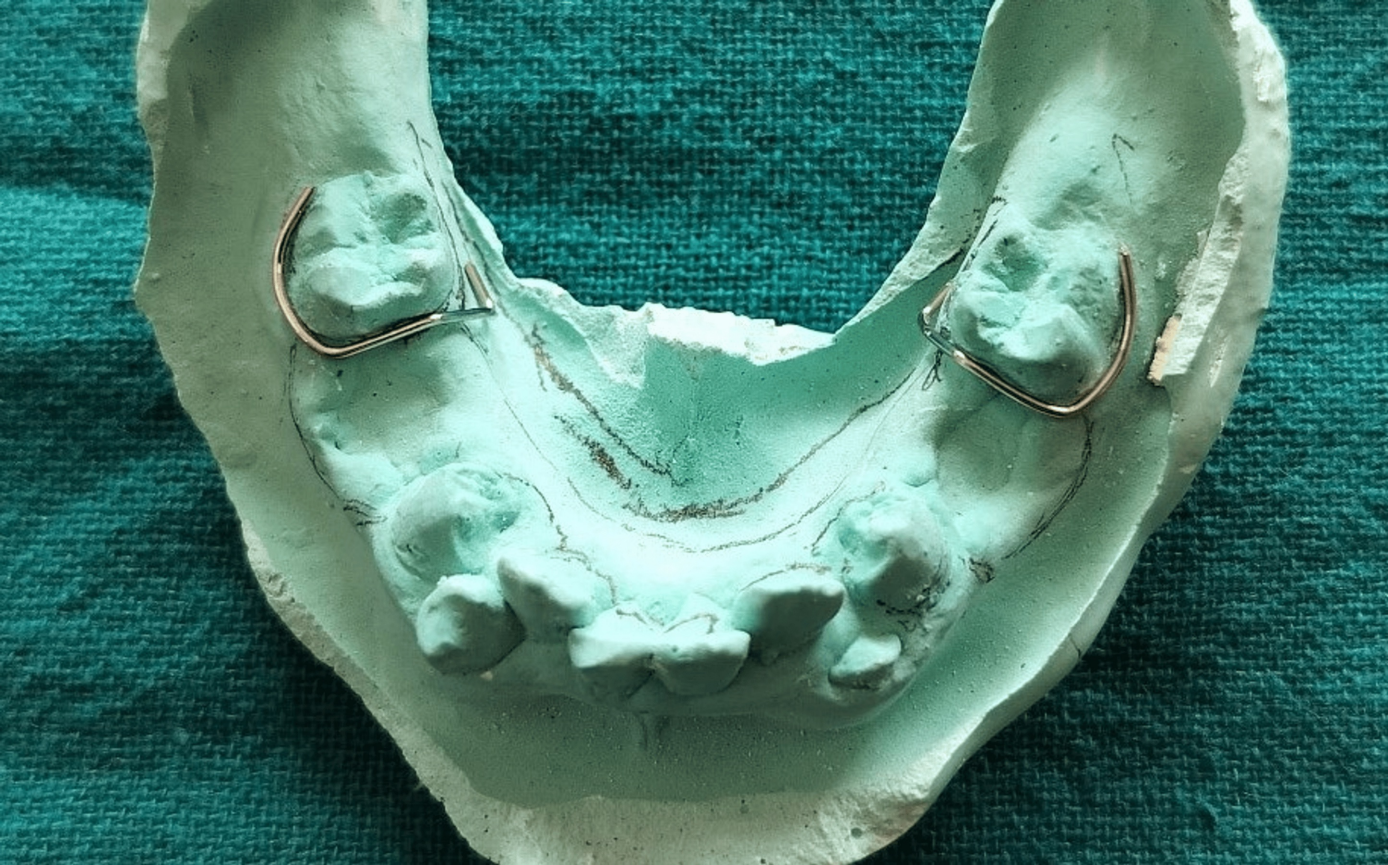
C-clasps fabricated on the working cast for teeth 36 and 46.

To allow time for the extraction sites to heal, the patient was recalled after four days, and alginate impressions of both arches were taken to obtain the working casts. On the mandibular working cast, C-clasps were designed for teeth 36 and 46 to provide stability (Figure 12).

**Figure 13:**
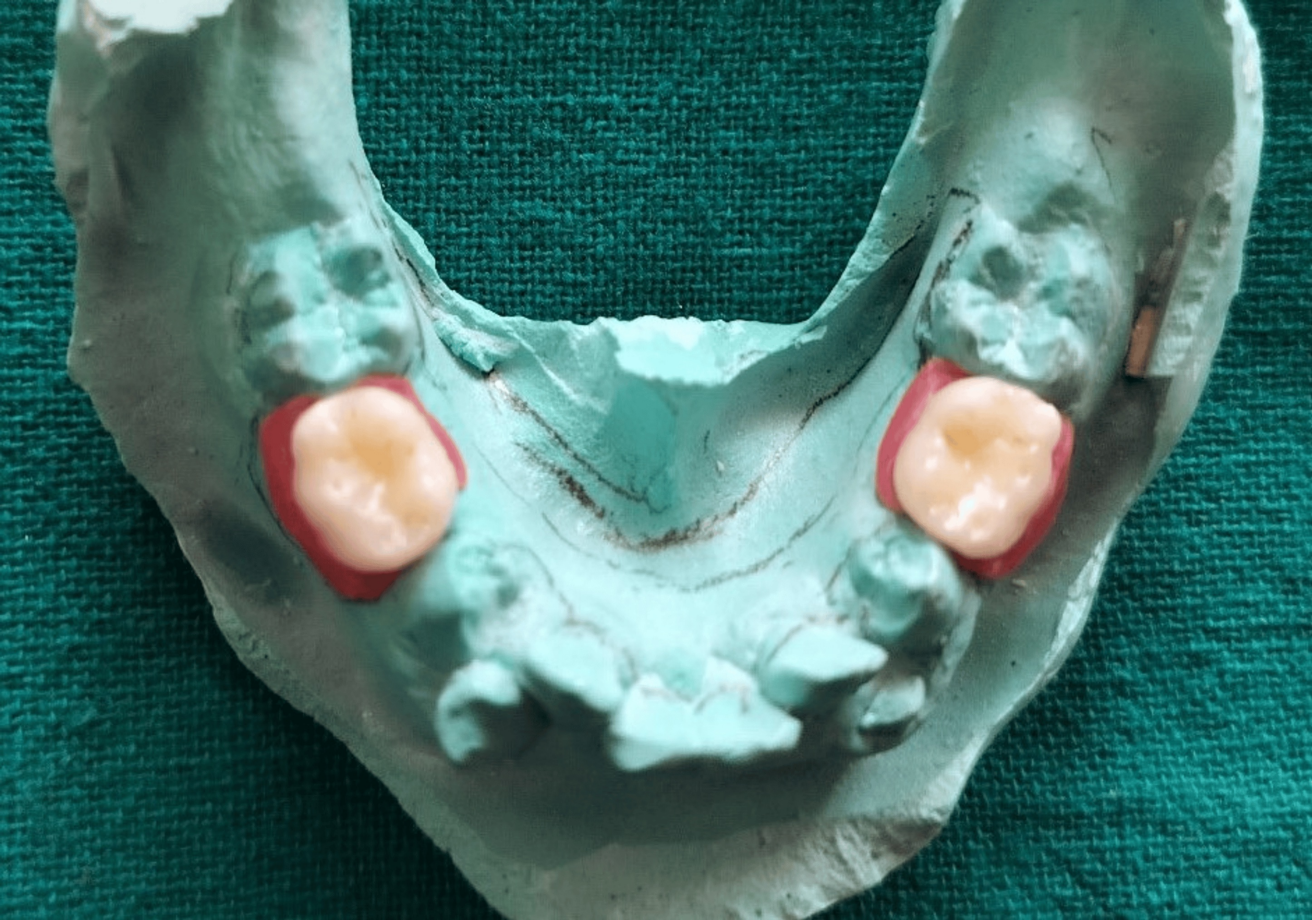
Decoronated teeth 75 and 85 secured on the cast using modeling wax to serve as pontics.

The extracted teeth, which had been stored in saline, were taken out and horizontally sectioned into coronal and radicular halves using a diamond disc bur, all under sterile conditions. After decoronating them to the cemento-enamel junction, the pulpal remnants were cleaned with hydrogen peroxide and sodium hypochlorite, and the teeth were then disinfected with glutaraldehyde. Following the same process as before, the teeth were smoothed at the cervical end and restored with composite. Finally, the trimmed teeth, 75 and 85, were attached to the cast with modeling wax, serving as pontics (Figure 13).

**Figure 14:**
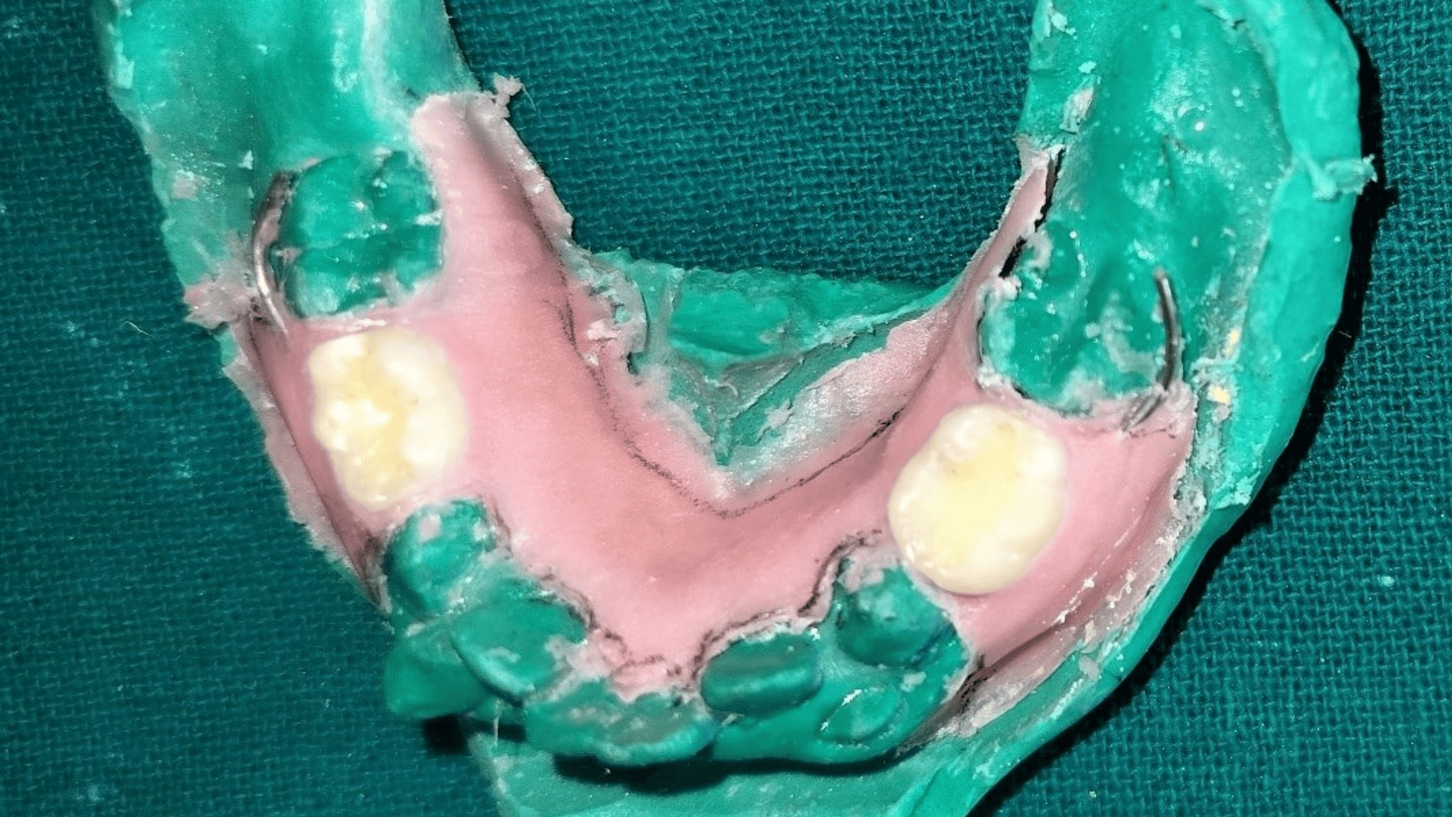
Appliance fabricated on the cast.

A biological removable functional space maintainer was subsequently created using self-cure acrylic resin, and occlusion was then checked and adjusted on the cast (Figure 14).

**Figure 15:**
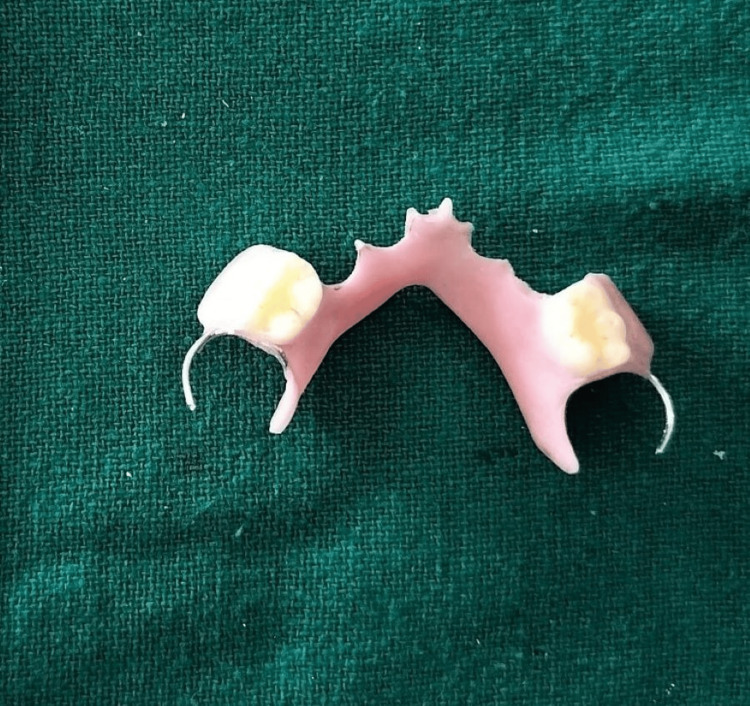
Trimmed, finished, and polished appliance.

The appliance was then trimmed, finished, and polished (Figure 15).

**Figure 16:**
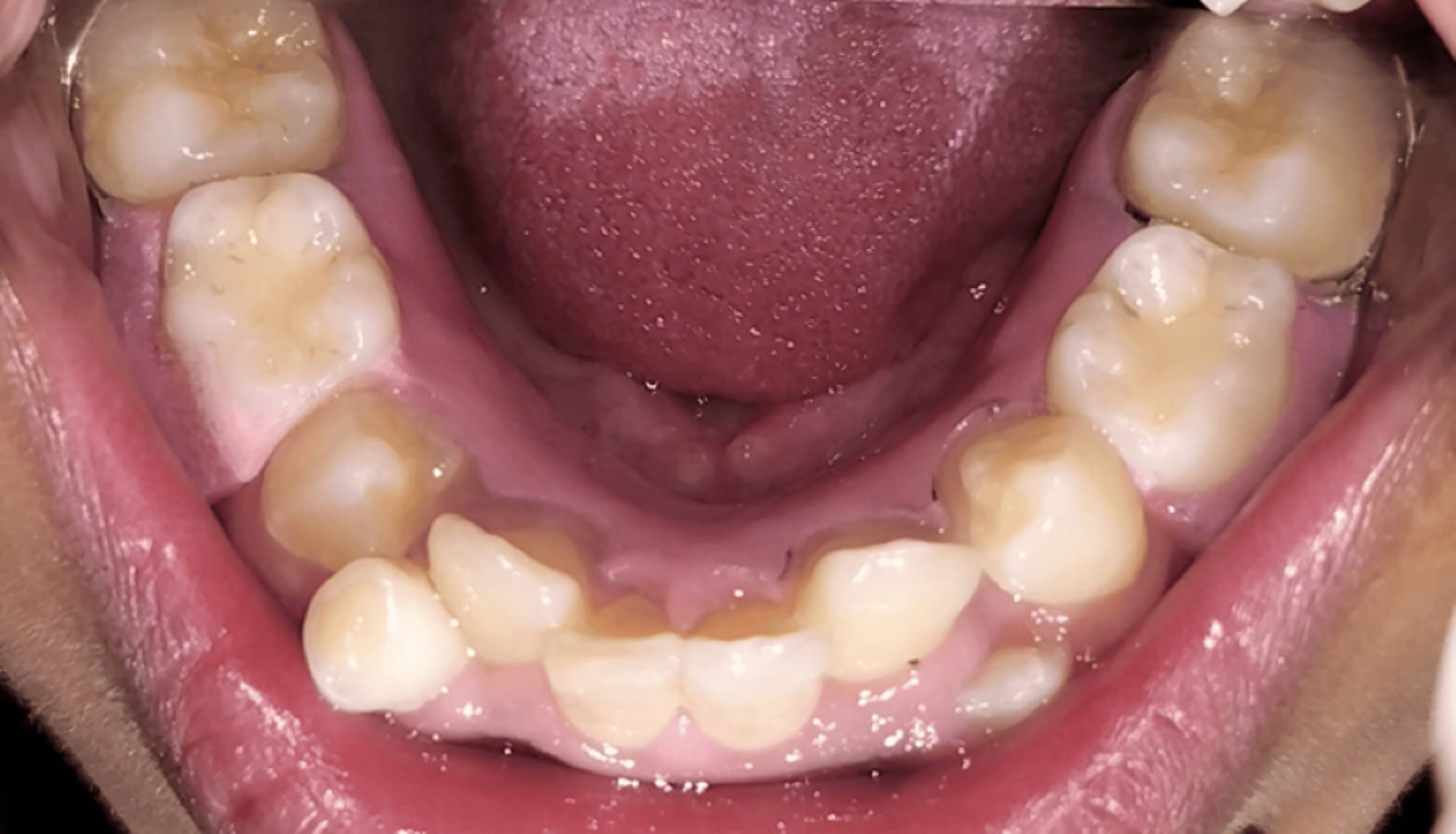
Post-insertion of biological removable functional space maintainer.

The patient was recalled the next day and the appliance was delivered. A thorough occlusion check was conducted, and comprehensive post-delivery instructions were provided (Figure 16).

**Figure 17:**
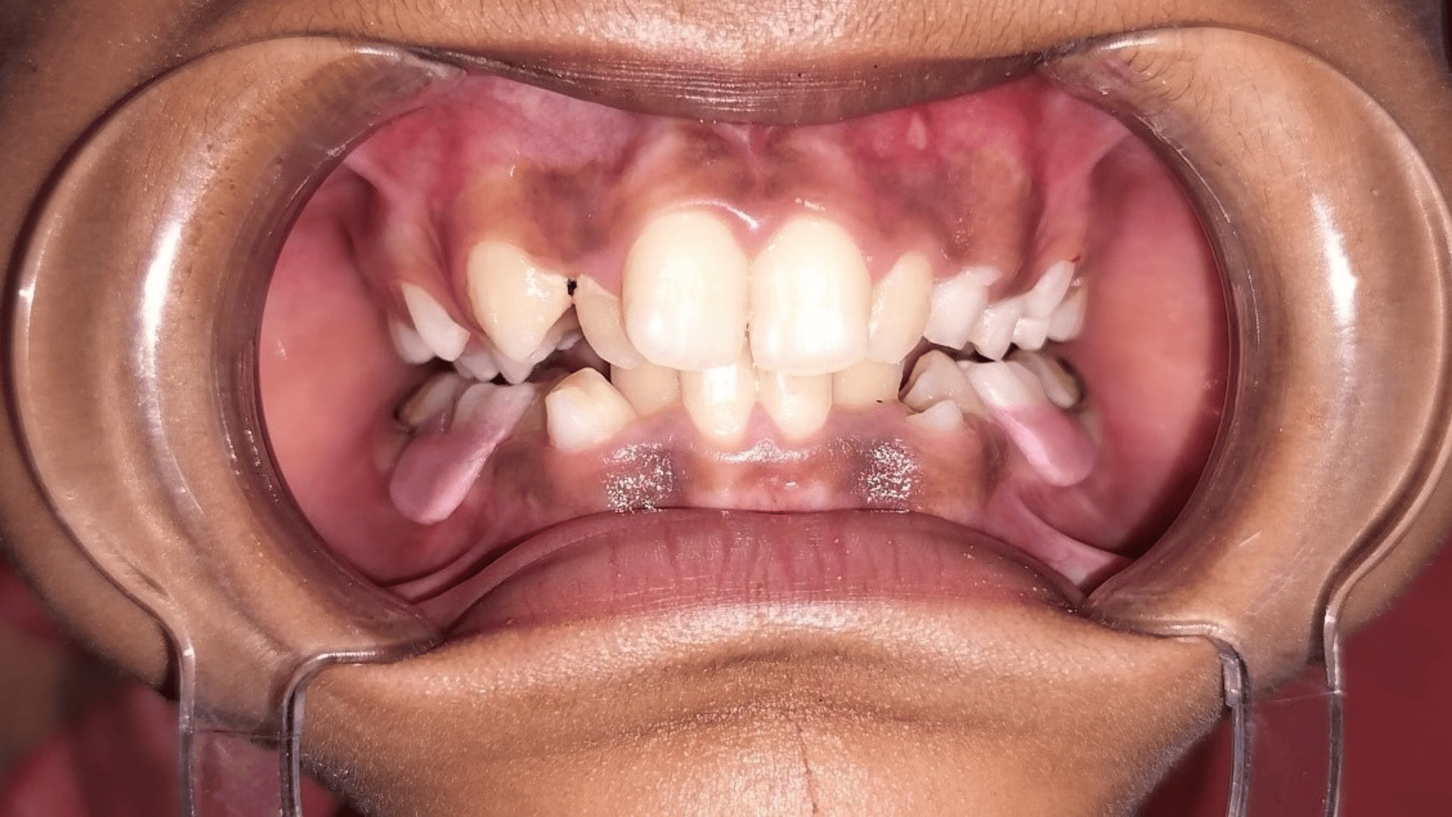
Photograph taken after one day.

After a one-day follow-up, the appliance demonstrated good retention (Figure 17).

**Figure 18:**
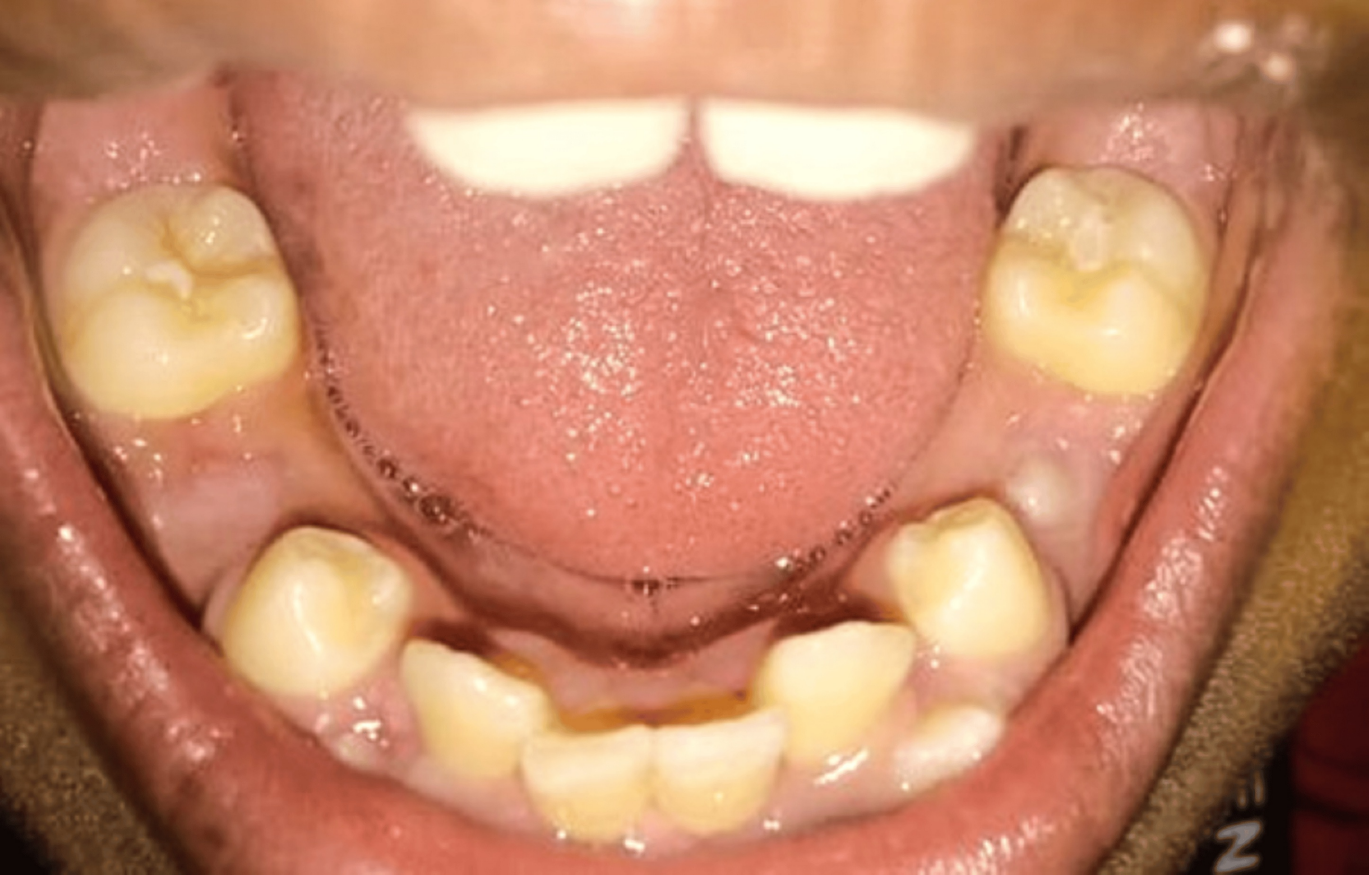
Follow-up examination showing eruption of tooth 35 and bulging of the alveolar ridge in relation to tooth 45, indicating the eruption process after the appliance is removed.

During a follow-up examination conducted after six weeks, erupting tooth 35 was clinically visible and a bulging over the alveolar ridge was seen in relation to tooth 45 (Figure 18).

Radiographic examination indicated approximately 0.5-1 mm of bone coverage over the unerupted tooth 45, suggesting that eruption is imminent. With the appliance fulfilling its intended purpose, the space maintainer was removed, and further orthodontic planning was initiated.

## Discussion

When dentures are crafted from the patient's own teeth, referred to as biologically based functional dentures or space maintainers, they offer significant benefits. These include minimal modification, practicality, cost-effectiveness, and outstanding aesthetics, as demonstrated in the initial case report [3]. It also offers the benefit of minimal occlusal adjustments [3] and delivers precise occlusion, as demonstrated in the second case report.

In mixed dentition, over-retained teeth can hinder or cause abnormal eruption of their successors, making early extraction crucial [4]. This issue was observed in the subsequent case report. To preserve space for the unerupted premolars and encourage their eruption, a biologically based denture was created through the integration of the natural extracted retained deciduous molars. Additionally, removable space maintainers help promote the eruption of permanent teeth by exerting pressure on the alveolar ridge [5] and also cause healthy stimulation of the mucosa to support the maintenance of alveolar bone and help in the same [6]. This was clearly demonstrated in our subsequent case report.

This technique of utilizing natural teeth is referred to as 'biological restoration,' a term introduced by Santos and Bianchi in 1991 [7]. Natural teeth play a crucial role in dental rehabilitation and function restoration. They improve aesthetics, display natural wear patterns, and have smooth surfaces that minimize biofilm accumulation and facilitate oral hygiene. Retaining a patient’s original crown is advantageous for addressing tooth loss, as it offers optimal options for size, alignment, color, and shape [8]. Incorporating natural teeth into a denture can potentially lead to discoloration or fractures. To prevent discoloration, it is important to promptly remove the pulp tissue, thoroughly clean the pulp chamber with normal saline, and store the teeth in a 100% humidity environment. Ensuring proper vertical and horizontal overlaps in the denture design helps maintain its stability and provides adequate protection for the natural teeth, reducing the risk of cracking or damage during use [9]. All these precautions were taken in our case series.

Lower anterior teeth are generally unsuitable for this technique due to insufficient retention at the cervical area and the potential for excessive weakening of the teeth after pulp removal. Similarly, this method is not advisable for posterior teeth because replacing their relatively large pulp chambers with autopolymerizing resin would not withstand masticatory forces over time [10]. However, in our case, where the application was intended for temporary use and short duration, it yielded favorable results. Therefore, case selection was carried out with careful consideration of all contraindications.

This approach is also contraindicated for mouth breathers, due to the loss of vitality and color in natural teeth from dryness and potential resin shrinkage, as well as bruxers, who exert higher biting forces, and patients with neuromuscular disorders [10]. In our cases, this issue did not arise; however, if accidental fracture or discoloration of a natural tooth were to occur, it could be replaced with an acrylic resin tooth modeled after the natural tooth or with an appropriately sized and colored artificial tooth [9].

## Conclusions

Prosthetic appliances play a crucial role in supporting dental and facial development in children. In two cases, natural teeth were incorporated into partial dentures: one to restore the aesthetics of anterior teeth, and the other to enhance chewing efficiency while maintaining space for and facilitating the eruption of unerupted teeth. Utilizing natural teeth not only ensured proper aesthetics and occlusion but also met patient expectations more efficiently and cost-effectively, and in less time.
